# Progressing Vulvar Melanoma Caused by Instability in cKIT Juxtamembrane Domain: A Case Report and Review of Literature

**DOI:** 10.3390/curroncol29050254

**Published:** 2022-04-29

**Authors:** Monika Englert-Golon, Bartłomiej Budny, Małgorzata Lewandowska, Bartosz Burchardt, Natalia Smolarek, Katarzyna Ziemnicka, Paweł Piotr Jagodziński, Marek Ruchała, Marlena Grabowska, Stefan Sajdak

**Affiliations:** 1 Division of Gynecological, Surgery Department of Gynaecology Obstetrics and Gynaecological Oncology, Poznan University of Medical Sciences, 60-535 Poznan, Poland; mal2015lewandowska@gmail.com (M.L.); panwieza@gmail.com (B.B.); smolarek.natalia@wp.pl (N.S.); marlenagrabowska@tlen.pl (M.G.); ssajdak@ump.edu.pl (S.S.); 2 Department of Endocrinology Metabolism and Internal Diseases, Poznan University of Medical Sciences, 60-356 Poznan, Poland; kaziem@ump.edu.pl (K.Z.); mruchala@ump.edu.pl (M.R.); 3 Department of Biochemistry and Molecular Biology, Poznan University of Medical Sciences, 60-781 Poznan, Poland; pjagodzi@ump.edu.pl; 4 Department of Forensic Medicine, Poznan University of Medical Sciences, 60-789 Poznan, Poland

**Keywords:** vulvar neoplasms, melanoma, recurrence, NGS

## Abstract

In order to identify the molecular pathways governing melanoma and track its progression, the next-generation sequencing (NGS) approach and targeted sequencing of cancer genes were employed. The primary tumor, as well as metastatic tissue, of an 84-year-old patient diagnosed with vulvar melanoma (VM), were investigated. The primary tumor specimen showed multiple somatic mutations in *TP53* gene, suggesting its major contribution to melanoma origin. The metastatic sample showed additional alterations, including other melanoma-related genes. Clinical relevancy is postulated to juxtamembrane region instability of *KIT* gene (c-KIT). We did not identify *BRAF* or *NRAS* alterations, which are typical for the most common melanoma pathway–MAPK cascade. However, it should be noted that this is the first report evidencing *PDGFRA* in melanoma, although its role in triggering VM needs to be further elucidated.

## 1. Introduction

Vulvar melanoma is a very rare tumor, and accounts for only 7–10% of all tumor lesions of the vulva [1,2]. Moreover, if we consider all melanomas occurring in the female genitals, most often they are localized at the vulva (1.3%) compared to the vagina (0.3%) [3,4]. To date, there is no consensus about the optimal management strategy of mucosal melanomas [5]. Future perspectives may arise from a deeper understanding of the molecular and biological mechanisms of melanomas, including microRNA expression, splicing and immunotype, in order to understand the other pathogenetic triggers and develop new target therapies [6,7]. The molecular pathways leading to development of the melanoma are complex, and encompass several various mechanisms (proliferative, senescence and apoptotic pathway), but a comprehensive elucidation of all mechanisms still represents a challenge for researchers [8]. The malignant transformation showed accumulation of genetic abnormalities that appear on certain stages and are different in regard to melanoma subtypes, thus necessitating application of targeted therapeutic strategies [9]. Cancer immunotherapy is currently paying particular attention to clinical oncologists due to recently reported efficacy and promising response [10,11]. The individual profile of every tumor is indicative for mapping of key players in order to obtain a comprehensive landscape of genetic changes, which would enhance the chances of efficient and successful targeted treatment [12]. Among the key mechanisms governing malignant transformation of melanocytes, the MAPK-ERK pathway represents the most common signaling cascade. This mechanism includes control of cell growth, proliferation and migration, and has been reported to play a major role in both the development and progression of melanoma. Therefore, the proliferative pathway encompasses the contribution of tyrosine kinases, often NRAS, but also BRAF, MEK1/2 and ERK1/2 proteins [13]. Cell senescence is another evidenced target mechanism triggering melanoma. The inhibition of telomere shortening, telomerase up-regulation, hyperactivity of MYC and ATM oncogenes, as well as p16-CDKN2A pathway (CDK4, CCND1, RB) is evidenced to promote uncontrolled proliferation and represents a common cancer cascade [14]. The diminishing abilities of inhibition for stimulating signaling consequently focus on the dysfunction of proapoptotic mechanisms and involvement of the p53-mediated cascade [15]. MDM2 protein interacts with p53 by blocking its activity and directing it towards immediate degradation. Consequently, signals that normally trigger apoptosis accumulate without any further programmed response. In conclusion, the variety of molecular pathways playing a role in different stages of melanomagenesis require a thorough molecular/genetic examination of tumor cells prior to making a crucial decision about application treatment protocols. The studies revealing crucial molecular triggers of the disease at different stages of melanoma neoplasm will facilitate selection of new-generation therapeutic strategies for cancer.

### Aim of the Research

The aim of the study was to present the clinical situation of patients diagnosed with vulvar melanoma and the identification of molecular pathways for this particular case. 

## 2. Materials and Methods

### 2.1. Clinical Examination

The biological material which was subjected to genetic analysis was collected from one of the patients, aged 84, treated in the Gynecology and Obstetrics clinical hospital of the Medical University in Poznan, Poland. The preoperative PET showed no distant lesions or changes in lymph nodes. Due to the lack of a sentinel node biopsy, to which the patient had not consented, clinical staging was performed instead of surgical. According to the AJCC and FIGO, it was IB. 

Four months after the primary radical surgery, the patient was again referred to the gynecological surgery department due to the observation of a nodular lesion located subcutaneously in the region of the urethral meatus. It was possible to reduce the tumor mass due to the inability to maintain the required margins of healthy tissue.

Due to the aggressive course of the disease and the poor general condition of the patient, it was decided to carefully examine the genetic changes in the preserved tissue samples from both operations, the results of which are presented in detail in the next paragraph.

### 2.2. Molecular Examination

Assessment of carcinogenesis was conducted using the NGS sequencing approach. DNA was extracted with High Pure FFPE DNA Isolation Kit (Roche Life Science, Basel, Switzerland) from FFPE melanoma specimens obtained during surgery. The genomic library for sequencing was prepared using Ion AmpliSeq^TM^ Library Kit 2.0. (Life Technologies, Carlsbad, CA, USA). DNA high-throughput sequencing was performed on the Ion Personal Genome Machine (PGM) Sequencer on the Ion 318 sequencing chip using Ion PGM Hi-Q View Sequencing Kit (Thermo Fisher, Waltham, MA, USA). The commercially available Cancer Comprehensive Panel (Thermo Fisher, USA) was used to study the coding regions and intronic flanking regions of the cancer related genes. The raw data obtained from genomic experiments were subjected for analysis using ION Reporter software. According to EMQN guidelines for an assessment of somatic variant detection in cancer, we determined a minimum of 500× sequence coverage of each variant. The mean sequencing coverage of the target regions across both samples was also >500-fold, and 97% of the target regions were covered. The obtained BAM/SAM files were subjected to a somatic mutation search using VariantCaller v5.2.1.38 software. Variants were assessed for its pathogenic potential and functional features of protein using FATHMM, MutationTaster2, Polyphen-2 and SIFT algorithms. We checked identified variants in the Catalogue of Somatic Mutations in Cancer (COSMIC) database, as well as population databases (dbSNP, GnomAD), to exclude common polymorphisms.

## 3. Results

After filtering data, we identified nine somatic mutations in five cancer genes (*PDGFRA, FBXW7, CSF1R, APC* and *TP53*). Mutations were present in various percentages, suggesting possible clonality. In the primary tumor tissue, we found eight somatic changes, of which seven variants were also presented in metastatic tissue (Table 1). One reported COSMIC variant (*PDGFRA*, COSM22413, p.V824V, c.2472C > T), was reported in population databases as a germline polymorphism; therefore, this change was discarded from further investigation, as well as similar variants. In the metastatic melanoma specimen, we identified additional 31 variants (Table 2). Among them, the majority represented known cancer hotspots, but thirteen were novel, thus-far unreported changes. All seven reported changes in a primary tumor were also detected in the metastatic sample with a higher percentage. Among newly identified genes, eleven other cancer targets were detected: *MPL*; *ERBB4*; *VHL*; *FGFR3*; *KIT*; *KDR*; *PTEN*; *KRAS*; *PTPN11*; *ERBB2;* and SMARCB1. All data encompassing mutations in primary and metastatic tissue, as well as their genomic coordinates, were included in Table 1 and Table 2.

## 4. Discussion

Vulvar melanoma is rarely detected in cases of young women. Similar to our patient, most of the cases described in the literature are postmenopausal patients. [16,17,18] At the diagnosis in our patient, the stage of the cancer was localized. Available literature data confirm the local progression of vulvar melanoma for about 65% of cases [3,19]. In the case of our patient, the vulvar melanoma was located in the right side of the frontal ventricle of the labia minora. According to the available data and literature, the labia minora and clitoris surroundings are the most common locations for VM to develop [20,21]. The starting point for vulvar melanoma can be characterized by pigmented and normal unchanged vulva skin [16,22]. Our patient had no previous signs and warts at the location of the vulvar tumor that could have been the starting point for the cancer. The literature describes three major types of vulvar cancer: superficial type, nodal form and vulvar mucosa [22,23,24] With clinical presentation, the molecular pathways governing melanoma development differ significantly. Recently, the three most common postulated pathways (proliferative, senescence and apoptotic) are characterized by different molecular triggers. Aulmann et al. reported the molecular characterization of 65 cases of vulvovaginal melanoma, finding no BRAF mutations but NRAS mutations and KIT amplifications in 12% of both vulvar and vaginal tumors. In agreement with these results, Rouzbauhman et al. found BRAF mutations in 8%, KIT mutations in 28%, NRAS mutations in 28% and TP35 mutations in 8% of vulvar tumors [5]. The signaling cascade starts at the cell membrane, either by the tyrosine kinase receptor (RTKs)-binding ligand or after the integrin adhesion to extracellular matrix, involving further RAS-GTPase action. In the described case, we did not note direct involvement of BRAF. The treatment strategy that shows good efficacy against BRAF altered melanomas (monotherapy with BRAF inhibitors vemurafenib, dabrafenib and encorafenib) [25]. However, this pathway cannot be excluded due to mutations in the platelet-derived growth factor receptor alpha (*PDGFRA*) gene. PDGFRA is a tyrosine kinase receptor, and phosphorylation substrates trigger activation of downstream pathways such as RAS-RAF-MEK-ERK (proliferation) and the PI3K-AKT-mTOR (survival) pathway. The alterations in *PDGFRA* are found explicitly in gastrointestinal stromal tumors (GIST) [26,27]. This is the first report evidencing PDGFRA in melanoma, although its role in triggering VM needs to be further elucidated. In the case under study, we also noted the presence of multiple alterations in *TP53* gene in a primary tissue, suggesting diminished proapoptotic abilities. This scenario, though less common, also cannot be excluded. The metastatic tissue showed further accumulation of somatic mutations and indicated another set of genes important for carcinogenesis. Among newly appearing factors playing a role in acquiring metastasis, tyrosine kinase receptor KIT (c-KIT) somatic mutations seem to be particularly important [28]. This subtype is shown to be common for mucosal melanomas and acral lentiginous melanomas, but its lower occurrence is demonstrated for all other melanomas. KIT is usually activated by an increase in the gene copy number and genomic amplification [29]. Promising results in treatment were found for activating point mutations (i.e., p.K642E and p.L576P variants), particularly those located in exon 11 and coding for the juxtamembrane domain. We detected a remarkable variability in this locus for metastatic tissue of a patient (deletion/insertion of amino acids at position 550–560). Moreover, our findings also confirm previous reports that BRAF (or NRAS) and c-KIT anomalies usually do not overlap [29,30]. Conversely, the c-KIT mutation spectrum overlaps with those found in a gastrointestinal stromal tumor (GIST) [31], and together with *PDGFRA* contribution (this case), a common mechanism of carcinogenesis is not excluded. In some cases, the *KIT* genotype of a primary lesion differs from its metastases [32]; that is an issue of the case under study, since we did not identify *KIT* changes in primary tissue. In therapy, c-KIT inhibitors were shown to have a positive response, particularly for neoplasms bearing c-KIT mutations compared with wild-type tumors [33,34]. The effective treatment would be of great importance here, since tumors with c-KIT anomalies are regarded to have a worse prognosis.

Among the truncating changes identified in other genes in metastatic tissue, we identified involvement of PTEN and APC. The activity of PTEN protein is evidenced to reduce PI3K (lipid phosphatase) [35,36,37,38], suppressing the activity of PI3K/AKT pathway. Inactivation of PTEN in human cancer results in upregulation of the AKT pathway (mainly AKT3 in melanoma) and its substrate mTOR, therefore mediating tumorigenesis [39,40,41,42]. Multiple truncating alterations in the PTEN gene have been found in various tumors, including lymphoma, thyroid, breast and prostate carcinomas, as well as melanoma. Elevated expression of AKT3 was found in 50% of dysplastic nevi, and 70% of primary or metastasizing melanomas. The *PTEN* gene is deleted in 30–40% of sporadic cases (with loss of the corresponding protein in 5–20% of primary melanomas) and in 30–50% of the cell lineages [43,44,45]. APC mediated pathway (β-Catenin/WNT signaling) could be another important factor to be considered as remarkable, also referring to an early stage of melanomagenesis. In our case, we identified truncating alterations in both primary and metastatic tissue. Cells with truncating APC rearrangements showed elevated expression levels of β-Catenin/T-cell factor (Tcf) target genes, disrupting stability and Tcf transactivation. In the absence of WNT-signals, β-catenin is targeted for degradation through phosphorylation controlled by a protein complex GSK3β)-axin-APC [46,47]. In vitro experiments evidenced that the suppression of APC transcripts of melanoma cells (hairpin RNAs) led to a Wnt signaling increase in cell proliferation, thus stabilizing levels of β-Catenin [48,49].

## 5. Conclusions

A tumor′s gene profile should be seen in the context of the genome inherited by a person. Individual genetic variation may define subgroups within a population as responding to therapy in different ways. It also seems important that the analysis of the genome throughout the course of the neoplastic disease could influence individual modifications of therapeutic strategies and initiate the development of gene-specific drugs.

## Figures and Tables

**Table 1:** Identified somatic variants in a primary melanoma tumor.

No.	Chr.	Genomic Pos hg19	Gene Symbol	Type	Amino Acid Alteration	Nucl. Ref.	Nucl. Alt.	Cosmic ID/dbSNP	HGVS
1	4	55141052	*PDGFRA*	deletion	p.S566Rfs*27	C	-	-	ENSP00000257290.5: p.Ser566ArgfsTer27
2	4	55141055	*PDGFRA*	deletion	p.P567Mfs*25	A	-	-	ENSP00000257290.5: p.Pro567MetfsTer25
3	4	153247278	*FBXW7*	SNV	intronic/splice site	T	C	rs147462419	-
4	5	112173894	*APC*	deletion	p.N869Ifs*47	A	-	-	ENSP00000257430.4: p.Asn869IlefsTer47
5	5	149453044	*CSF1R*	SNV	p.L301*	A	T	rs121913390, COSV53841262, COSV53842469	ENSP00000286301.3: p.Leu301Ter
6	17	7573993	*TP53*	deletion	p.N345Mfs*25	T	-	COSV53589460	ENSP00000269305.4: p.Asn345MetfsTer25
7	17	7578450	*TP53*	SNV	p.M160I	C	A	COSV52849333, COSV53297171, COSV53424484, COSV53438638	ENSP00000269305.4: p.Met160Ile
8	17	7579373	*TP53*	deletion	p.G105Afs*18	C	-	rs1567555907, COSV52708806, COSV52766179, COSV52793434	ENSP00000269305.4: p.Gly105AlafsTer18

**Table 2:** Identified somatic variants in a metastatic melanoma tumor.

No.	Chr.	Genomic Position hg19	Gene Symbol	Type	Amino Acid Alteration	Nucl. Ref.	Nucl. Alt.	Cosmic ID/dbSNP	HGVS
1	1	43815009	*MPL*	SNV	p.W515L	G	T	rs121913615, COSV65243776, COSV65245195	ENSP00000361548.3: p.Trp515Leu
2	2	212652796	*ERBB4*	SNV	p.P150P/splice	T	C	rs1450712101	ENSP00000342235.4: p.Pro170Pro
3	3	10183815	*VHL*	SNV	p.P95R	C	G	CM092616, COSV56544941, COSV56556284, COSV56567454	ENSP00000256474.3: p.Pro95Arg
4	3	10188260	*VHL*	deletion	p.L135Yfs*24	T	-	-	ENSP00000256474.3: p.Leu135TyrfsTer24
5	3	10188297	*VHL*	deletion	p.F148Lfs*11	T	-	rs869025653, CM982009	ENSP00000256474.3: p.Phe148LeufsTer11
6	4	1808398	*FGFR3*	SNV	p.C719S	G	C	-	ENSP00000339824.4: p.Cys721Ser
7	4	55593594	*KIT*	deletion/insertion	p.E550K*10	G	-	COSV55411322	ENSP00000288135.6: p.Glu554LysfsTer10
8	4	55593597	*KIT*	SNV	p.V551I	G	A	COSV55405668	ENSP00000288135.6: p.Val551Ile
9	4	55593597	*KIT*	Deletion/insertion	p.V551Yfs*9	G	-	COSV55405668	ENSP00000288135.6: p.Val555TyrfsTer9
10	4	55593601	*KIT*	deletion and insertion	p.W557Gfs*7	T	-	CM005329, COSV55386440, COSV55387014, COSV55389479	ENSP00000288135.6: p.Trp557GlyfsTer7
11	4	55593610	*KIT*	SNV	p.V559A	T	C	rs121913517, CM013551, COSV55386973, COSV55388782, COSV55393324 COSM1255	ENSP00000288135.6: p.Val559Ala
12	4	55962445	*KDR*	SNV	p.G893G/splice	A	G	-	NP_002244.1: p.Gly893Gly
13	4	55980239	*KDR*	SNV	intronic/splice site	C	T	rs7692791	ENST00000263923.5: c.798 + 54G > A
14	5	112175378	*APC*	insertion	p.S1364Kfs*11	A	AA	COSV57337694, COSV57379285	ENSP00000257430.4: p.Ser1364LysfsTer11
15	5	112175408	*APC*	deletion	p.P1373Lfs*42	C	-	COSV57387710, COSV57395032	ENSP00000257430.4: p.Pro1373LeufsTer42
16	5	112175600	*APC*	deletion	p.T1438Hfs*35	A	-	COSV57401545	ENSP00000257430.4: p.Thr1438HisfsTer35
17	5	112175622	*APC*	deletion	p.T1445Qfs*28	A	-	-	ENSP00000257430.4: p.Thr1445GlnfsTer28
18	5	112175756	*APC*	deletion	p.L1489Yfs*18	T	-	COSV57327796	ENSP00000257430.4: p.Leu1489TyrfsTer18
19	5	112175761	*APC*	deletion	p.F1491Lfs*16	T	-	-	ENSP00000257430.4: p.Phe1491LeufsTer16
20	5	112175766	*APC*	deletion	T1493Rfs*14	C	-	COSV57375363	ENSP00000257430.4: p.Thr1493ArgfsTer14
21	5	112175772	*APC*	deletion	S1495Vfs*12	A	-	-	ENSP00000257430.4: p.Ser1495ValfsTer12
22	10	89685271	*PTEN*	insertion	p.L57Ffs*6	T	TT	COSV64290332	ENSP00000361021.3: p.Leu57PhefsTer6
23	10	89685289	*PTEN*	deletion and insertion	p.N63Tfs*36	A	-	rs1554897267, COSV64298134	ENSP00000361021.3: p.Asn63ThrfsTer36
24	10	89720804	*PTEN*	insertion	p.T319Nfs*6	A	AA	rs786204892, CD972424	ENSP00000361021.3: p.Thr319AsnfsTer6
25	10	89720812	*PTEN*	deletion	p.N323Mfs*21	A	-	rs121913291	ENSP00000361021.3: p.Asn323MetfsTer21
26	12	25378647	*KRAS*	SNV	p.K117N	T	G	rs770248150, COSV55504752, COSV55545304	ENSP00000256078.5: p.Lys117Asn
27	12	112926961	*PTPN11*	SNV	intronic/splice site	C	T	-	ENSP00000489597.1: p.Arg531Arg
28	17	7578280	*TP53*	deletion	p.P190Lfs*57	G	-	CM161004, COSV52664064, COSV52987047 COSV53313892	ENSP00000269305.4: p.Pro190LeufsTer57
29	17	7579472	*TP53*	SNV	p.P72H	G	T	rs1042522, CM961374, COSV52666208, COSV53098660	ENSP00000269305.4: p.Pro72His
30	17	37881001	*ERBB2*	SNV	p.V777A	T	C	-	ENSP00000269571.4: p.Val777Ala
31	22	24133954	*SMARCB1*	SNV	p.Y35Y/splice	C	T	rs1176990918, CM110285	ENSP00000340883.6: p.Tyr35Tyr
